# Mitigating the impact of mechanisms causing neuronal degeneration

**DOI:** 10.1002/nep3.41

**Published:** 2024-03-19

**Authors:** Xunming Ji, Piotr Walczak, Johannes Boltze

**Affiliations:** ^1^ Beijing Institute of Brain Disorders Capital Medical University Beijing China; ^2^ Center for Advanced Imaging Research, Department of Diagnostic Radiology and Nuclear Medicine University of Maryland Baltimore Maryland USA; ^3^ School of Life Sciences University of Warwick Coventry UK

**Keywords:** Alzheimer's disease, extracellular vesicles, intracranial pressure, microglia, neurodegeneration, stem cells, stress, stroke, vascular dementia

1

Primary and secondary neurodegeneration is a pathological hallmark of numerous central nervous system (CNS) disorders. Although many mechanisms leading to neurodegeneration are well understood, previous approaches aiming at providing protection from neurodegeneration were often futile. A potential explanation may be that recent research discovered additional pathomechanisms leading to neurodegeneration. Thus, simply targeting single neurodegenerative mechanisms may only have minor therapeutic impact. Addressing multiple neurodegenerative mechanisms may be a more viable strategy. Moreover, the restoration of lost brain tissue turned out to be a very complex endeavor.^1^ Despite making some initial progress with the use of biocompatible scaffolds and hydrogels,^2^ the goal of meaningful restoration remains elusive so far. It is therefore essential to obtain a detailed and holistic understanding of neurodegenerative mechanisms to continuously improve our strategies to mitigate their impact in CNS disorders. This is particularly important for major diseases such as stroke, Alzheimer's disease, and vascular dementia, as they are leading contributors to severe disabilities, cognitive dysfunction, loss of quality of life, and patient mortality worldwide. This issue of *Neuroprotection* presents original contributions and reviews providing novel insights into mechanisms of primary and secondary neurodegeneration. Those may represent therapeutic targets for novel approaches to provide neuroprotection. This editorial highlights some intriguing aspects found within the articles published in the current issue of *Neuroprotection*.

Haupt et al. review the role of microglia in ischemic stroke. As the resident CNS immune cells, microglia become activated after an ischemic event and contribute to pro‐inflammatory signaling in early stages following stroke. They attract peripheral immune cells to the lesion site and facilitate ischemic lesion organization by removing cell debris. Microglia also exert anti‐inflammatory functions, contributing to the resolution of neuroinflammation in subacute and chronic stages. However, microglia can contribute to secondary neuronal damage and cell loss when remaining in the pro‐inflammatory activation state over extended time. Thus, a good balance between the pro‐inflammatory and anti‐inflammatory microglia polarization states is important for a good outcome after stroke. In their work, Haupt et al. explain microglia polarization including mixed phenotypes forming a spectrum in between the pro‐ and anti‐inflammatory types. They also review in detail how microglia polarization can be beneficially modulated by stem cells and extracellular vesicles.

Alzheimer's disease is among the most relevant neurodegenerative disorders. The often rapid decline in cognitive capabilities puts a significant burden to patients, their relatives and healthcare systems. Established treatments of Alzheimer's disease such as cholinesterase inhibitors, NMDA receptor antagonists and, more recently, monoclonal antibodies directed against the pathognomonic amyloid β plaques only have a minor therapeutic impact. The need for novel therapeutic approaches has therefore been a recurring topic in *Neuroprotection*.^3^, ^4^ Zhu et al. review the potential use of repetitive transcranial magnetic stimulation (rTMS) in Alzheimer's disease. The procedure is safe, noninvasive, and has been used in clinical trials on other CNS disorders including stroke. The authors start by providing a detailed overview of technical parameters of rTMS such as stimulus frequency, intensity, patterns, and coils. They also provide a thorough review of the clinical trials and report its impact on different brain areas such as the prefrontal cortex, the precuneus cortex, as well as on the cerebellum and explain novel rTMS application protocols. A major part of the review by Zhu et al. elucidates potential mechanisms of rTMS in Alzheimer's disease. They present evidence of neural network modulation and enhancement of synaptic plasticity by rTMS. rTMS was also shown to reduce neuronal apoptosis and to promote hippocampal neurogenesis. It is further believed that it can exert beneficial effects via microglia and astrocytes. Since many of the mechanistic investigations reviewed were conducted in animal models of Alzheimer's disease, the translatability of these findings to human patients remains to be shown.

The work by Wang et al. focuses on cerebral glutamate metabolism in the context of vascular dementia. They provide a comprehensive review covering types of vascular dementia, current treatment options, and animal models of vascular dementia. They then focus on glutamate as the most important excitatory neurotransmitter. Based on the explanation of the glutamate metabolism in the steady state the authors review all aspects of glutamate excitotoxicity including glutamate release and recovery as well as the role of glutamate receptors. Wang et al. also briefly discuss ideas how manipulation of the glutamate metabolism may help in the treatment of vascular dementia.

Peng and coworkers focused on experimental autoimmune encephalomyelitis (EAE), a commonly applied animal model for multiple sclerosis. Both EAE and multiple sclerosis are characterized by an immune response against myelin that initially leads to oligodendrocyte death but also neuronal injury and neurodegeneration in later stages. Peng et al. investigated the role of interleukin (IL)‐17‐secreting CD8^+^ T‐cytotoxic (Tc17) cells in the pathogenesis of EAE as previous studies predominantly focused on IL‐17‐secreting CD4^+^ T‐helper (Th17) cells. The response as well as the production of pro‐inflammatory cytokines such as IL‐17 and interferon‐γ were weaker in Tc17 cells compared to Th17 cells. Nevertheless, EAE scores of mice in which EAE was induced by the adoptive transfer of Tc17 cells were similar to EAE scores in mice that received Th17 cells. These findings shed first light on the role of Tc17 cell in the pathogenesis of EAE, but additional research will be required to provide a more comprehensive picture. Moreover, the role of Tc17 cells in multiple sclerosis needs to be elucidated in future translational studies.

An increase in intracranial pressure (ICP) is often reported after major ischemic strokes being complicated by edema buildup. However, ICP elevation has also been reported after minor stroke not being accompanied by a major edema.^5^ The mechanisms behind this phenomenon are incompletely understood. The increase of ICP can reduce cerebral perfusion and collateral blood flow what in turn could have detrimental impact on the penumbra after ischemic stroke, exacerbating expansion of the infarct core. An elevated ICP after minor strokes has been reported in rats and larger mammals. Warren et al. now show that ICP also increases in mice after photothrombotic stroke, and the kinetics of the increase is similar to that observed in other species. Although this finding may appear minor at first glance, it is of high experimental relevance because mice are most frequently used in stroke research. Thus, further studies can investigate the mechanisms of post‐stroke ICP elevation in more detail using mice.

In summary, the present issue of *Neuroprotection* features interesting research reporting neurodegenerative mechanisms in the mammalian brain and provides suggestions on how this can be addressed in future neuroprotective approaches. Reported investigations span from molecular and cellular mechanisms to an overview of ongoing clinical activities, thus comprising all aspects of translational neuroscience.

## AUTHOR CONTRIBUTIONS

All authors listed have conceptualized, written and edited the publication, and approved its final version for submission.

## CONFLICTS OF INTEREST STATEMENT

Xunming Ji, Piotr Walczak, and Johannes Boltze are Co‐Editors in Chief of *Neuroprotection*. They were blinded from reviewing or making decisions on the manuscript. The article was subject to the journal's standard procedures, with peer review handled independently of the Co‐Editors in Chief and their research groups. P.W. is a founder and holds equity IntraArt, LLC. Ti‐com, LLC.

## ETHICS STATEMENT

Not applicable.

## Data Availability

Not applicable.
